# Acute lymphoblastic leukemia: a comprehensive review and 2017 update

**DOI:** 10.1038/bcj.2017.53

**Published:** 2017-06-30

**Authors:** T Terwilliger, M Abdul-Hay

**Affiliations:** 1 New York University School of Medicine, New York, USA; 2 Department of Hematology, New York University Perlmutter Cancer Center, New York, USA

## Abstract

Acute lymphoblastic leukemia (ALL) is the second most common acute leukemia in adults, with an incidence of over 6500 cases per year in the United States alone. The hallmark of ALL is chromosomal abnormalities and genetic alterations involved in differentiation and proliferation of lymphoid precursor cells. In adults, 75% of cases develop from precursors of the B-cell lineage, with the remainder of cases consisting of malignant T-cell precursors. Traditionally, risk stratification has been based on clinical factors such age, white blood cell count and response to chemotherapy; however, the identification of recurrent genetic alterations has helped refine individual prognosis and guide management. Despite advances in management, the backbone of therapy remains multi-agent chemotherapy with vincristine, corticosteroids and an anthracycline with allogeneic stem cell transplantation for eligible candidates. Elderly patients are often unable to tolerate such regimens and carry a particularly poor prognosis. Here, we review the major recent advances in the treatment of ALL.

## Introduction

Acute lymphoblastic leukemia (ALL) is a malignant transformation and proliferation of lymphoid progenitor cells in the bone marrow, blood and extramedullary sites. While 80% of ALL occurs in children, it represents a devastating disease when it occurs in adults. Within the United States, the incidence of ALL is estimated at 1.6 per 100 000 population.^1^ In 2016 alone, an estimated 6590 new cases were diagnosed, with over 1400 deaths due to ALL (American Cancer Society). The incidence of ALL follows a bimodal distribution, with the first peak occurring in childhood and a second peak occurring around the age of 50.^2^ While dose-intensification strategies have led to a significant improvement in outcomes for pediatric patients, prognosis for the elderly remains very poor. Despite a high rate of response to induction chemotherapy, only 30–40% of adult patients with ALL will achieve long-term remission.^3^

## Pathophysiology

The pathogenesis of ALL involves the abnormal proliferation and differentiation of a clonal population of lymphoid cells. Studies in the pediatric population have identified genetic syndromes that predispose to a minority of cases of ALL, such as Down syndrome, Fanconi anemia, Bloom syndrome, ataxia telangiectasia and Nijmegen breakdown syndrome.^4, 5, 6, 7^ Other predisposing factors include exposure to ionizing radiation, pesticides, certain solvents or viruses such as Epstein-Barr Virus and Human Immunodeficiency Virus.^8, 9, 10^ However, in the majority of cases, it appears as a de novo malignancy in previously healthy individuals. Chromosomal aberrations are the hallmark of ALL, but are not sufficient to generate leukemia. Characteristic translocations include t(12;21) [*ETV6-RUNX1*], t(1;19) [*TCF3-PBX1*], t(9;22) [*BCR-ABL1*] and rearrangement of *MLL*.^11^ More recently, a variant with a similar gene expression profile to (Philadelphia) Ph-positive ALL but without the BCR-ABL1 rearrangement has been identified. In more than 80% of cases of this so-called Ph-like ALL, the variant possesses deletions in key transcription factors involved in B-cell development including IKAROS family zinc finger 1 (IKZF1), transcription factor 3 (E2A), early B-cell factor 1 (EBF1) and paired box 5 (PAX5).^12^ Similarly, kinase-activating mutations are seen in 90% of the Ph-like ALL. The most common of these include rearrangements involving ABL1, JAK2, PDGFRB, CRLF2 and EPOR, activating mutations of IL7R and FLT3 and deletion of SH2B3, which encodes the JAK2-negative regulator LNK.^13^ This has significant therapeutic implications as it suggests that Ph-like ALL, which tends to carry a worse prognosis, may respond to kinase inhibitors. In fact, Roberts *et al.*^14^ showed that cell lines and human leukemic cells expressing ABL1, ABL2, CSF1R and PDGFRB were sensitive *in vitro* and *in vivo* human xenograft models to second-generation TKIs (for example, dasatinib.); those with EPOR and JAK2 rearrangements were sensitive to JAK kinase inhibitors (for example, ruxolitinib); and those with ETV6-NTRK3 fusion were sensitive to ALK inhibitors crizotinib. Furthermore, Holmfeldt *et al.*^15^ recently described the genetic basis of another subset with poor outcomes, hypodiploid ALL. In near-haploid (24–31 chromosomes) ALL, alterations in tyrosine kinase or Ras signaling was seen in 71% of cases and in IKAROS family zinc finger 3 (IKZF3) in 13% of cases. In contrast, low-hypodiploid (32–39 chromosomes) ALL, alterations in p53 (91%), IKZF2 (53%) and RB1 (41%) were more common. Both near-haploid and low-hypodiploid exhibited activation of Ras- and PI3K-signaling pathways, suggesting that these pathways may be a target for therapy in aggressive hypodiploid ALL.^15^

Most of the clinical manifestations of ALL reflect the accumulation of malignant, poorly differentiated lymphoid cells within the bone marrow, peripheral blood, and, extramedullary sites. Presentation can be nonspecific, with a combination of constitutional symptoms and signs of bone marrow failure (anemia, thrombocytopenia, leukopenia). Common symptoms include ‘B symptoms’ (fever, weight loss, night sweats), easy bleeding or bruising, fatigue, dyspnea and infection. Involvement of extramedullary sites commonly occurs and can cause lymphadenopathy, splenomegaly or hepatomegaly in 20% of patients.^16, 17^ CNS involvement at time of diagnosis occurs in 5–8% of patients and present most commonly as cranial nerve deficits or meningismus.^3^ T-cell ALL also may present with a mediastinal mass.

Diagnosis is established by the presence of 20% or more lymphoblasts in the bone marrow or peripheral blood.^16^ Evaluation for morphology, flow cytometry, Immunophenotyping and cytogenetic testing is valuable both for confirming the diagnosis and risk stratification. Lumbar puncture with CSF analysis is standard of care at the time of diagnosis to evaluate for CNS involvement. If the CNS is involved, brain MRI should be performed. Other evaluation includes complete blood count with differential and smear to evaluate the other hematopoietic cell lines, coagulation profiles and serum chemistries. Baseline uric acid, calcium, phosphate and lactate dehydrogenase should be recorded to monitor for tumor lysis syndrome.

## Classification

The first attempt at classifying ALL was the French American British (FAB) morphological criteria that divided ALL into 3 subtypes (L1, L2 and L3) based on cell size, cytoplasm, nucleoli, vacuolation and basophilia.^18^ In 1997, the World Health Organization proposed a composite classification in attempt to account for morphology and cytogenetic profile of the leukemic blasts and identified three types of ALL: B lymphoblastic, T lymphoblastic and Burkitt-cell Leukemia.^19^ Later revised in 2008, Burkitt-cell Leukemia was eliminated as it is no longer seen as a separate entity from Burkitt Lymphoma, and B-lymphoblastic leukemia was divided into two subtypes: B-ALL with recurrent genetic abnormalities and B-ALL not otherwise specified. B-ALL with recurrent genetic abnormalities is further delineated based on the specific chromosomal rearrangement present (Table 1).^20^ In 2016, two new provisional entities were added to the list of recurrent genetic abnormalities and the hypodiploid was redefined as either low hypodiploid or hypodiploid with TP53 mutations.^21^ In adults, B-cell ALL accounts for ~75% of cases while T-cell ALL comprises the remaining cases.

## Prognostic factors

Accurate assessment of prognosis is central to the management of ALL. Risk stratification allows the physician to determine the most appropriate initial treatment regimen as well as when to consider allogeneic stem cell transplantation (Allo-SCT). Historically, age and white blood cell count at the time of diagnosis have been used to risk stratify patients. Increasing age portends a worsening prognosis. Patients over the age of 60 have particularly poor outcomes, with only 10–15% long-term survival.^22^ Age is at least in part a surrogate for other prognosticators as the elderly tend to have disease with intrinsic unfavorable biology (for example, Philadelphia chromosome positive, hypodiploidy and complex karyotype), more medical comorbidities and inability to tolerate standard chemotherapy regimens but helps guide therapy nonetheless. In the largest prospective trial to determine optimal treatment, MRC UKALL XII/ECOG E2993 found a significant difference of disease-free (DFS) and overall survival (OS) based on age using a cutoff of 35 in Ph-negative disease.^23^ Similarly, they found an elevated white blood cell count at diagnosis, defined as >30 × 10^9^ for B-ALL or >100 10^9^ for T-ALL, was an independent prognostic factor for DFS and OS. On the basis of these results, Ph-negative disease could be categorized as low risk (no risk factors based on age or WBC count), intermediate risk (age >35 or elevated WBC count), or high risk (age >35 and elevated WBC count). The 5-year OS rates based on these risk categories were 55, 34 and 5%, respectively.^23^

Although clinical factors play an important role in guiding therapy, cytogenetic changes have a significant role in risk determination. The cytogenetic aberration with the greatest impact on prognosis and treatment is the presence of the Philadelphia chromosome, t(9;22). The prevalence of t(9;22) in adult ALL can range from 15–50% and increases with age.^24^ Ph-positivity has implications both in terms of prognosis and for treatment. Historically, Ph-positive ALL has a 1-year survival of around 10%. However, with the development of TKIs, survival has improved and thus the Ph-status of all patients must be obtained prior to starting therapy. Subsequent analysis of MRC UKALL XII/ECOG E2993, identified cytogenetic subgroups of Ph-negative disease with inferior outcomes. These included t(4;11), *KMT2A* translocation, t(8;14), complex karyotype (⩾ 5 chromosomal abnormalities) and low hypodiploidy (30–39 chromosomes)/near triploidy (60–78 chromosomes). In contrast, patients with hyperdiploidy and del(9p) had a significantly better outcome.^25^ In a later study, the Southwest Oncology Group (SWOG) showed that among the 200 study patients, cytogenetic profile was a more important prognostic factor than age or WBC count.^26^ More recently, a subset of high-risk ALL without t(9;22) has been identified with a genetic profile similar to that of Ph-positive ALL. This so called, Ph-like ALL has been associated with poor response to induction chemotherapy, elevated minimal residual disease and poor survival.^13, 14, 27^

In addition to disease characteristics at the outset, it has long been recognized that response to initial therapy predicts outcome. Historically, treatment response was evaluated morphologically. Recently, it has become standard practice to evaluate patients for minimal residual disease (MRD) using molecular techniques such as flow cytometry and PCR.^28^ Several studies have shown the importance of MRD in assigning risk.^29, 30, 31, 32, 33, 34^ Bruggemann *et al.*^29^ re-stratified standard-risk patients to low risk, intermediate risk and high risk with relapse rates of 0%, 47% and 94%, respectively, based on the persistence of elevated MRD, defined as >10^−4^. In a multivariate analysis of 326 adolescent and adult patients with high-risk Ph-negative ALL treated in The Programa Espanol de Tratamientos en Hematologia (PETHEMA ALL-AR-03), Ribera *et al.*^35^ showed that poor MRD clearance, defined as levels >1 × 10^−3^ after induction and levels >5 × 10^−4^ after early consolidation by flow cytometry, was the only significant prognostic factor for disease-free and overall survival.

On the basis of what is known about prognostic factors in adult ALL, the National Comprehensive Cancer Network (NCCN) has developed recommendations to approach risk stratification.^16^ The National Cancer Institutes defines adolescent and young adults (AYA) to be those aged 15–39 years. The NCCN recognizes that AYA may benefit from treatment with pediatric-inspired regimens and thus are considered separately from adults >40 years.^36, 37^ Both age groups are then stratified into high-risk Ph-positive and standard-risk Ph-negative subgroups. The Ph-negative subgroup can further be categorized as high-risk based on the presence of MRD, elevated WBC (defined above) or unfavorable cytogenetics (defined above).

## Established treatments

The structure of treatment of adult ALL has been adapted from pediatric protocols. Unfortunately, while long-term survival approaches 90% for standard-risk pediatric ALL, the success rate is much more modest in adults. Chemotherapy consists of induction, consolidation and long-term maintenance, with CNS prophylaxis given at intervals throughout therapy. The goal of induction therapy is to achieve complete remission and to restore normal hematopoiesis. The backbone of induction therapy typically includes vincristine, corticosteroids and an anthracycline.^38, 39^ In the Cancer and Leukemia Group B 8811 trial, Larsen *et al.*^40^ achieved a complete response rate of 85% and a median survival of 36 months. The 4-week long induction schedule consists of cyclophosphamide on day 1, 3 consecutive days of daunorubicin, weekly vincristine, biweekly l-asparaginase and 3 weeks of prednisone.^40^ Due to high induction-related mortality, one-third dose reductions of cyclophosphamide and daunorubicin were implemented for patients older than 60 and the duration of prednisone was shortened to 7 days in this age group. The role of L-asparaginase, while standard in pediatric protocols, is a challenge in adults at times due to the increased rate of adverse events.^41^ In fact, in the UKALL 14 Trial, Patel *et al.*^42, 43^ demonstrated that asparaginase toxicity was the leading cause of induction-related mortality and the protocol was amended to omit asparaginase for patients over the age of 40. The MRC UKALL XII/ECOG 2993^23^ regimen utilizes a similar structure to CALGB 8811. Induction is divided into two phases of four weeks. In contrast to CALGB 8811, cyclophosphamide is omitted in phase I of induction, but a single dose of intrathecal methotrexate is added for CNS prophylaxis. In phase II of induction, cyclophosphamide is introduced along with cytarabine, oral 6-mercaptopurine (6-MP), four additional intrathecal doses of methotrexate, and cranial radiation if CNS is positive. After induction therapy, patients received three cycles of intensification therapy of methotrexate with leucovorin rescue and l-asparaginase. Eligible patients with high-risk disease and a matched donor, then underwent Allo-SCT. All others were randomized to standard consolidation/maintenance or autologous stem cell transplant. This study yielded a complete response rate of 91% and an overall 5-year survival of 38%.^23^

The Hyper-CVAD (HCVAD)/ Methotrexate-cytarabine regimen is utilizes an alternative structure to the approaches described above. It consists of four cycles of hyperfractionated cyclophosphamide, vincristine, doxorubicin and dexamethasone alternated with four cycles of high dose cytarabine and methotrexate.^44^ CNS prophylaxis with 4-16 doses of intrathecal chemotherapy depending on predetermined risk of CNS disease. HCVAD has demonstrated similar efficacy to the ECOG trial with a 92% complete response rate and 32% 5-year disease-free survival.^44^ Several studies have suggested a benefit to using dexamethasone as opposed to prednisone due to the ability of dexamethasone to achieve higher concentrations in the CNS. Despite a reduction in CNS relapse and improved event-free survival, dexamethasone has increased risk of adverse events compared to prednisone. Since there have been no studies comparing overall survival, the benefit of one corticosteroid over the other has not been established.^45, 46^

After induction, eligible patients may go on to Allo-SCT while all others go on to intensification/consolidation and maintenance.^47^ Consolidation varies in the different protocols, but generally utilize similar agents to induction and includes intrathecal chemotherapy and cranial radiation for CNS prophylaxis at times. Maintenance therapy consists of daily 6-MP, weekly methotrexate, and vincristine and a 5-day prednisone pulse every 3 months. Maintenance is administered for 2–3 years after induction, beyond which it has not been shown to have benefit.^17, 47^

Special consideration must be made in the treatment of Ph-positive ALL. Historically, Ph-positive ALL was a very bad player with 5-year survival ~5–20% and Allo-SCT being the only chance for cure.^48, 49^ Various studies have found that matched-sibling Allo-SCT may improve long-term survival to 35–55%, however, availability of matched donors represents a significant limitation.^49, 50, 51^ The advent of TKIs marked a turning point in the treatment of Ph-positive ALL. Thomas *et al.*^52, 53^ showed that when added to traditional HCVAD, imatinib resulted in improvement in 3-year OS (54 vs 15%). Despite these promising results, some patients fails treatment due to resistance or relapse, particularly in the CNS where imatinib has limited penetration.^54^ Second-generation ABL kinase inhibitor, dasatinib, was developed as a dual src/abl kinase inhibitor for chronic myeloid leukemia with a superior resistance profile to imatinib. Dasatinib was also shown to penetrate the blood-brain barrier and was effective at treating CNS disease in a mouse model and pediatric Ph-positive ALL.^55^ In the first study of dasatinib in Ph-positive ALL, Ravandi *et al.*^56^ found a CR rate of 96% when dasatinib was combined with HCVAD, and a 5-year OS of 46%. In a subsequent, multi-center trial HCVAD plus dasatinib achieve a 3-year OS of 71% in adult patients younger than 60.^57^ In addition, prior resistance to imatinib did not preclude a response to dasatinib.^58^ In addition, dasatinib was shown to be effective in inducing complete remission when used in combination with prednisone and intrathecal methotrexate.^59^ In the GIMEMA LAL1205 study,^59^ it was noted that the most common cause of relapse was a T315I mutation in the ABL kinase doman. Ponatinib, a third-generation TKI with the ability to inhibit most BCR-ABL1 kinase domain mutations, has recently gained approval for resistant Ph-positive ALL. The PACE trial^60^ demonstrated the ability of ponatinib to generate a cytogenetic response in 47% of Ph-positve ALL patients after dasatinib failure. When compared head-to-head with dasatinib, ponatinib achieved significantly better 3-year EFS and OS when used as frontline therapy.^42, 61, 62^ These data suggest that ponatinib may soon have a role in the frontline therapy of Ph-positive ALL.

Recent studies have suggested that the AYA population, defined as aged 15–39, may benefit from treatment on pediatric-inspired protocols. In an analysis of 262 AYA patients aged 16–21 on pediatric protocol CCG 1961, Nachman *et al.*^63^ reported a 5-year EFS of 68%. Furthermore, patients in the study that were treated on augmented intensity therapy performed better. In a prospective study, Stock *et al.*^64^ treated 317 patients aged 17–39 on Children’s Oncology Group AALL0232 protocol. Median EFS approached 60 months, which was statistically higher than the null hypothesis of 32 months. OS at 2-years was 78%.^64^ Similarly, The Group for Research on Adult Acute Lymphoblastic Leukemia (GRAAL), compared 225 patients up to the age of 60 who were treated on pediatric-inspired regimen and historical data from 712 adults treated on standard adult regimen LALA-93.^36^ They observed a significant improvement in CR, EFS and OS, which was most marked in patients younger than age of 45 years. In fact, in patients older than 45 years, there was a significantly higher rate of chemotherapy-related events compared to younger patients, suggesting that an age cutoff for pediatric-inspired regimens is appropriate. However still one of the adult regimens is still considered for AYA patients is HCVAD±rituximab. An MD Anderson Cancer Center study revealed no significant difference in CR rate or OS in AYA patients treated with HCVAD±rituximab vs an augmented-Berlin-Frankfurt-Munster regimen.^65^

## Refractory/relapsed disease

While 85–90% of patients go into remission after induction therapy, there are subsets that are refractory to induction therapy. In addition, a majority of patients that do achieve CR go on to relapse. Options of salvage therapy for relapsed/refractory (r/r) Ph-negative disease include augmented cytotoxic chemotherapy, reformulated single-agent chemotherapy and novel monoclonal antibodies. Augmented-HCVAD for salvage therapy was inspired by pediatric regimens that employ intensified doses of vincristine, corticosteroids and asparaginase in frontline therapy. Faderl *et al.*^66^ treated 90 patients (median age 34) with relapsed or refractory disease with HCVAD in which the dosing of vincristine, dexamethasone and asparaginase where intensified as follows: vincristine 2 mg i.v. weekly on days 1, 8 and 15; dexamethasone 80 mg i.v. or orally (p.o.) on days 1–4 and 15–18, and pegaspargase 2500 units/m^2^ i.v. on day 1 of the hyper-CVAD courses (1, 3, 5 and 7) and day 5 of the methotrexate/cytarabine courses (2, 4, 6 and 8). The majority of patients were in first salvage and ten patients were primary refractory, and patients with prior exposure to HCVAD were not excluded. Complete response was observed in 47% of the patients, with a median duration of 5 months. Median DFS and OS were 6.2 and 6 months respectively.^66^ It was also noted that the addition of rituximab to HCVAD for B-ALL with high CD20 expression to improve the activity of this salvage regimen.

In patients with relapsed/refractory ALL, particularly those with multiple relapses, toxicity of multi-agent cytotoxic therapy may be limiting. Therefore, attempts have been made at salvage therapy with a single agent. In subgroup analysis of 70 patients receiving second salvage therapy with a single agent (most commonly vinorelbine (6), clofarabine (5), nelarabine (4) and topotecan (4)), only 3 achieved a complete response.^67, 68^ Vincristine sulfate liposomes injection (VSLI) was developed to overcome the dosing and pharmacokinetic limitations of nonliposomal vincristine (VCR). In a phase II study in adults with Ph-negative ALL in their second or greater relapse, VSLI was administered weekly at a dose of 2.25 mg/m^2^.^69^ Of the 65 adults enrolled, 20% achieved complete response with a median duration of 23 weeks (range 5–66). Twelve patients were bridged to Allo-SCT, with five long-term survivors.^69^ This study led to the accelerated approval of VSLI for salvage therapy in 2012. VSLI was well tolerated with a side effect profile similar to standard-formulation VCR, despite the massive cumulative doses of VCR achieved.

Despite the modest ability of cytotoxic chemotherapy to prolong survival, the only hope for long-term survival in these regimens remains Allo-SCT. However, recently novel monoclonal antibodies have transformed the landscape of salvage therapy by offering a chance at cure may be without Allo-SCT. The first of these is the bispecific anti-T-cell receptor/anti-CD19 antibody, blinatumomab. The proposed mechanism of action of blinatumomab is that it engages T cells to activate a B-cell specific inflammatory and cytolytic response.^70^ Blinatumomab was first studied in patients with MRD positive ALL. In one trial, 80% of patients became MRD negative after the first cycle of blinatumomab, with 60% of patients remaining in CR at a median follow-up of 33 months.^71^ Importantly, in a multi-center trial (BLAST), Gokbuget *et al.*^72^ confirmed the ability of blinatumomab to eliminate MRD and showed no difference in OS or relapse-free survival (RFS) between patients who received Allo-SCT during the first CR (CR1) and those who did not. Based on these results, blinatumomab was studied for relapsed/refractory Ph-negative ALL. The landmark study was a multi-center, single-arm, open-label phase 2 trial in which 189 patients with primary refractory and relapsed ALL received single-agent therapy with blinatumomab. CR was achieved after 2 cycles in 43 with 82% achieving MRD negativity. The median response duration and the overall survival were 9 and 6 months, respectively.^73^ Based on these results, blinatumomab was approved by the FDA for relapsed and refractory ALL in 2016. Subsequently, blinatumomab was compared to investigator’s choice of chemotherapy for r/r Ph-negative ALL in the phase 3 randomized trial (TOWER study). The blinatumomab study group (*n*=271) had a median survival of 7.7 months (95% confidence interval (CI): 5.6, 9.6) versus 4.0 months (95% CI: 2.9, 5.3) for standard of care (*n*=134) (*P*=0.012, hazards ratio (HR), 0.71).^74^ The study was terminated early for efficacy based on these results. Blinatumomab has also been investigated for r/r Ph-positive disease. In the ALCANTARA trial, standard dose blinatumomab was given for up to 5 cycles in 45 patients. CR was observed in 36 and 88% of whom were MRD negative, and with a median follow-up of 9 months, the median OS was 7.1 months.^75^ Future investigation is planned for the frontline use of blinatumomab for Ph-positive ALL in conjunction with TKIs.^76^ The toxicity profile of blinatumomab is acceptable. The most frequent adverse events include fever, chills, neutropenia, anemia and hypogammaglobulinemia.^3^ More significant adverse events are rare, but include cytokine release syndrome, altered mental status and seizures.^73^ Death from sepsis that is thought to be treatment-related has been reported.

Frontline therapy is the same for B-cell ALL and T-cell ALL. However, owing to different biology of the two subtypes, T-cell ALL is not amenable to salvage treatment with blinatumomab. Fortunately, alternative options for salvage therapy exist. Nelarabine is a T-cell specific purine nucleoside analog that is FDA approved for r/r T-cell ALL. Nelarabine accumulates in T cells at a high rate and incorporates into DNA causing an inhibition of DNA synthesis and subsequent apoptosis.^77^ In a phase 2, open-label, multi-center trial, nelarabine was administered on alternate day schedule (days 1, 3 and 5) at 1.5 g/m^2^/day for r/r T-cell ALL. Cycles were repeated every 22 days. The rate of complete remission was 31% (95% CI, 17, 48%), the median DFS and OS were 20 weeks with a 1-year OS of 28%.^77^ However, there is still more that needs to be done to achieve a better response and overall survival in patients with relapsed/refractory B- and T-cell ALL.

## Future therapies

### 1-Monoclonal antibodies

#### A-CD22-Directed therapy

CD22 is a B-lineage differentiation antigen expressed in B-cell ALL in 50–100% of adults and 90% of children.^78, 79, 80^ Upon binding of an antibody, CD22 is rapidly internalized, thus making it an attractive target for delivering immunotoxin to leukemic cells.^81^

#### Epratuzumab

Epratuzumab is an unconjugated monoclonal antibody targeting CD22 that has been studied in pediatric and adult relapsed/refractory ALL. Epratuzumab was evaluated in 15 pediatric patients as part of a salvage therapy regimen. The antibody was administered as a single-agent followed by the antibody in combination with standard re-induction chemotherapy. The treatment resulted in a CR in 9 of the patients, with 7 achieving complete MRD clearance at the end of re-induction.^82^ A phase 2 study in adults with relapsed/refractory disease evaluated the addition of epratuzumab to clofaribine/cytarabine. The study demonstrated a superior response rate when compared to historical data of clofaribine/cytarabine alone.^83^ More recently, epratuzumab conjugated to the topoisomerase I inhibitor, SN-38, has been shown to have activity against B-cell lymphoma and leukemia cell lines in *in vitro* and *in vivo* preclinical studies.^84^

#### Inotuzumab ozogamicin

Inotuzumab ozogamicin (InO) is a monoclonal antibody against CD22 that is conjugated to calicheamicin, a potent cytotoxic compound that induces double-strand DNA breaks.^85^ Upon internalization of the immunoconjugate, calicheamicin binds DNA and causes double-stranded DNA breaks, which induces apoptosis. Preclinical studies showed that calicheamicin conjugated to an anti-CD22 antibody resulted in potent cytotoxicity leading to regression of B-cell lymphoma and prevention of xenograft establishment at picomolar concentrations.^86^ Phase 1 studies in non-hodgkin lymphoma (NHL) established a maximum tolerated dose of 1.8 mg/m^2^ InO given intravenously every 3 to 4 weeks.^87^ Subsequently, InO was studied in adults with relapsed/refractory ALL.^88^ In this phase 2 trial, 90 patients were treated with either a single infusion every 3 to 4 weeks or weekly InO infusions. Cumulative doses were equivalent among the two treatment strategies. Overall response rate was 58%, with similar response between the two dosing schedules. Median survival was 6.2 months, with a non-significant benefit seen in weekly dosing. However, toxicity was greatly improved by weekly dosing, with a significant reduction in fever, hepatotoxicity and veno-occlusive disease.^89^ A second phase 2 study of 35 patients with CD22+ ALL in second salvage or later showed similar complete response rate (66%) and median overall survival (7.4 months).^90^ Based on these results, Kantarjian *et al.*^91^ compared weekly dosing of InO to standard chemotherapy for relapsed/refractory ALL. The rate of complete remission was significantly higher in the InO group versus standard chemotherapy 80.7% (95% CI, 72.1–87.7) vs 29.4% (95% CI, 21.0–38.8), *P*<.001).^91^ Progression-free survival (5 months vs 1.8 months) and overall survival (7.7 months vs 6.7 months) were also significantly prolonged with InO compared to standard chemotherapy. The most common adverse events of InO treatment included thrombocytopenia and neutropenia. Veno-occlusive liver disease occurred in 11% of patients treated with InO compared to 1% of those receiving standard chemotherapy.^91^ Based on these results, InO was granted Breakthrough Therapy status by the FDA in 2015 and is a strong candidate for expedited approval for relapsed/refractory ALL.

InO has also been studied in frontline therapy in combination with low-intensity HCVAD for elderly patients >60 years.^92^ These patients are prone to adverse events from chemotherapy and have poorer outcomes than their younger counterparts. In attempt to reduce toxicity, doxorubicin was eliminated from induction therapy, and cyclophosphamide, prednisone, methotrexate and cytarabine were given at reduced doses. InO was given during each of the first four courses. The regimen was well tolerated and produced superior 1-year OS as compared to historical data among similar patient population (78 vs 60%).^92^

#### Moxetumomab pasudodotox

A third anti-CD22 monoclonal antibody, moxetumomab, is currently in development for treatment of pediatric and adult ALL. Moxetumomab is a reformulation of an older study drug, BL22, which was composed the variable region (F_v_) of an anti-CD22 monoclonal antibody fused to *Pseudomonas aeruginosa* exotoxin A.^93^ BL22 was shown to be highly active against Hairy Cell Leukemia in a phase 2 trial.^94^ In a phase 1 trial of children with relapsed/refractory ALL, BL22 was well tolerated and exhibited anti-leukemic activity at all doses, but clinical benefits were transient and modest.^95^ Therefore, BL22 was reformulated as moxetumomab to contain a F_v_ fragment with greater affinity for CD22. In phase 1 trials, moxetumomab showed an overall activity rate of 70% in children with relapsed/refractory ALL.^96^ Enrollment is ongoing for a phase 1/2 trial of moxetumomab pasudodotox for treatment of relapsed/refractory ALL in adults.^97^

#### Combotox

Combotox is a combination immunotoxin that contains a 1:1 mixture of anti-CD19 and anti-CD22 antibodies, both conjugated to the cytotoxin deglycosylated ricin-A chain. In pediatric patients with relapsed/refractory ALL, combotox led to a CR in 3 of 17 patients. In addition, six additional patients experienced a >95% reduction in peripheral blasts.^98^ In adults with relapsed/refractory disease, combotox led to reduction of peripheral blasts in all patients; however, a durable response was not seen as blast count rebounded quickly after the final dose of combotox.^99^ A phase I trial is recruiting patients to evaluate combotox in combination with cytarabine for adults with relapsed/refractory ALL (NCT01408160).

#### B-CD20

CD20 is a B-lineage specific antigen expressed at nearly all stages of differentiation on the surface of both normal and malignant B-cells. Signaling through CD20 plays a role in cell cycle progression, differentiation pathways and regulation of apoptosis. CD20 is expressed in 40–50% of precursor lymphoblasts, and confers a worse prognosis.^100^ Moreover, CD20-positive leukemia responds poorly to dose intensification, highlighting the need for targeted therapy. The addition of rituximab, a first-generation anti-CD20 monoclonal antibody, has improved outcomes in these patients, but resistance to rituximab represents a limitation to its use.

#### Ofatumumab

Ofatumumab is a second-generation anti-CD20 antibody with a distinct binding site from that of rituximab. Ofatumumab was first showed to have benefit in fludarabine-refractory chronic lymphocytic leukemia, irrespective of prior rituximab exposure.^101^ Ofatumumab induces higher levels of complement-dependent cytotoxicity (CDC) and has a slower dissociation rate than rituximab, and thus holds promise for CD20+ lymphoid malignancies both as frontline therapy and as salvage for rituximab-refractory disease.^102, 103^ In a phase 2 study, ofatumumab was used in combination with HCVAD in patients with either newly diagnosed pre-B CD20+ ALL or those who had completed a single course of chemotherapy. In all study patients, CD20+ expression was >1%.^104^ Ofatumumab was administered at a dose of 2 grams on days 1 and 11 of the first 4 cycles of induction therapy. All but one patient (98%) achieved CR after cycle 1 and 93% of patients were negative for MRD at end induction. The 3-year CR and OS rates were 78% and 68%, respectively.^105^ This is similar to benefits seen when rituximab was used as frontline therapy in CD20+ ALL.^106^ Ofatumumab represents a potential alternative frontline therapy for CD20+ pre-B-ALL and an option for patients who failed a rituximab-based regimen.

#### Obinutuzumab

Another novel anti-CD20 monoclonal antibody, obinutuzumab, has shown promise in preclinical trials for CD20-positive B-ALL. Obinutuzumab was engineered to have enhanced affinity for the FcγRIIIa receptor on effector cells and thus enhanced antibody-dependent cell-mediated cytotoxicity (ADCC).^107^ This compromises the ability of obinutuzumab to activate complement and predictably, CDC was inferior to that of rituximab and ofatumumab *in vitro*. However, obinutuzumab induced direct cell death and ADCC more rapidly and effectively. When all three mechanisms of cell death were evaluated together in B-cell depletion assays, obinutuzumab was more effective than either rituximab or ofatumumab achieving higher maximal depletion and lower EC_50_. Furthermore, obinutuzumab was superior in inhibiting growth in NHL xenograft models.^107^ Awasthi *et al.*^108^ compared obinutuzumab to ritixumab in pre-B-ALL cell lines and found obinutuzumab to be superior in inducing cell death and ADCC. In a pre-B-ALL xenograft model, overall survival was improved with obinutuzumab compared to ritixumab.^108^ In clinical trials, obinutuzumab has been added to chlorambucil for treatment of adults with CLL and shown to prolong progression-free survival and improve complete response rate when compared to rituximab and chlorambucil.^109^ Taken together, these results suggest a role for obinutuzumab in CD20+ pre-B-ALL.

#### REGN1979

REGN1979 is a biallelic monoclonal antibody targeting CD20 and CD3. The theory of REGN1979 is similar to that of blinatumumab, to engage T cells and B-cells thus resulting in activation of T-cell immune response against B-cells. REGN1979 prevented the establishment of lymphoma xenografts and led to complete tumor regression in murine models.^110^ In addition, in a primate model, REGN1979 led to a complete and durable depletion of B-cells. When compared to treatment with rituximab, treatment with REGN1979 led to significantly more profound depletion of B-cells.^110^ The safety of REGN1979 was established in a phase 1 trial of 25 patients with NHL and CLL. Dose-dependent antitumor activity was observed. The most significant adverse events include cytokine release syndrome (CRS) and hypotension.^111^ A phase 2 trial of REGN1979 in relapsed/refractory ALL is currently open for recruitment (NCT02651662).

#### C-CD19

CD19 is the most widely expressed B-lineage specific antigen, expressed during all stages of differentiation, but lost on maturation to plasma cells. CD19 serves as a co-receptor for the B-cell surface immunoglobulin and its activation triggers a phosphorylation cascade involving src-family kinases and PI3K as well as the activation of c-myc, leading to proliferation and differentiation.^112, 113, 114^ CD19 is expressed in nearly all B-cell leukemias, and is rapidly internalized upon binding of an antibody, making it an ideal candidate for immunoconjugate therapy.^115^

#### Coltuximab ravtansine (SAR3419)

Coltuximab ravtansine is an anti-CD19 humanized monoclonal antibody conjugated to a semisynthetic maytansinoid compound, an anti-tubulin molecule similar to vincristine. Maytansinoids are more potent than vinca alkaloids, and thus have been of limited use in systemic therapy due to unacceptable toxicity.^116^ However, this potency makes them attractive candidates for targeted delivery. In preclinical studies, SAR3419 monotherapy delayed progression in pre-B-ALL xenografts and provided objective response. When used in combination with a chemotherapy regimen that mimicked pediatric induction protocols, SAR3419 was effective at prolonging the duration of remission.^117^ SAR3419 was then evaluated in a Phase 1 clinical trial with CD19+ B-cell lymphoma. Dose-limiting toxicities were reversible blurred vision and neuropathy. A maximum tolerated dose (MTD) of 160 mg/m^2^ administered once every three weeks was established. Reduction of tumor size was seen in 74% of patients, including 47% of patients with rituximab-resistant disease.^118^ An initial phase 2 clinical trial was terminated early due to low response rate of 25%.^119^

#### Denintuzumab mafodotin (SGN-CD19A)

A second anti-CD19 conjugated monoclonal antibody, denintuzumab mafodotin, is currently in development. In this case, the antibody is linked to the microtubule-disrupting agent monomethyl auristatin F (MMAF). In a phase 1 study of patients with relapsed/refractory B-ALL or aggressive B-cell lymphomas, a complete response rate of 35% was observed.^120^ Dosing interval of 3 weeks was shown to be superior to weekly dosing. An MTD was identified at 5 mg/kg q3wk. Interestingly, among Ph-positive B-ALL, the response rate was 63%, leading to recruitment of Ph-positive patients for an expansion cohort. These results warrant further evaluation of denintuzumab mafodotin for relapsed/refractory ALL.

#### ADCT-402

ADCT-402 is the newest anti-CD19 monoclonal antibody to enter development. It is a humanized monoclonal antibody conjugated to a pyrrolobenzodiazepine (PBD). PBDs are a class of natural antibiotics derived from actinomycetes bacteria that inhibit cell division by binding in the minor groove of DNA and cross-linking strands of DNA. *In vivo* studies show superior antitumor activity of ADCT-402 against CD19-positive lymphoma than maytansinoid or auristatin based therapy.^121^ A phase 1 trial of ADCT-402 for relapsed/refractory ALL is underway (NCT02669264).

#### D-CD25

CD25 is a cell surface antigen and component of the Interleukin-2 receptor (IL-2 R) heterotrimer.^122^ Binding of IL-2 R by its ligand activates JAK/STAT, MAP kinase and phosphoinositide 3-kinase (PI3K) signaling pathways, leading to cell proliferation. IL-2 R is rapidly recycled upon binding of its ligand.^123^ The IL-2 R signaling pathway is particularly activated in T-cell immune response, and has thus been an attractive target for post-transplant immunosuppression. In some studies, CD25 expression has been as high as 30% of pre-B-ALL lymphoblasts, including 100% expression among the Ph-positive subset.^124^

#### ADCT-301

ADCT-301 is a monoclonal antibody against CD25 conjugated to a PBD. In preclinical studies, ADCT-301 has been shown to be potently cytotoxic to CD25-positive anaplastic large cell lymphoma and Hodgkin lymphoma cell lines. *In vivo*, ADCT-301 exhibited antitumor activity in xenograft and disseminated mouse models.^122^ A phase 1 trial is recruiting participants for ADCT-301 in relapsed/refractory AML and ALL (NCT02588092).

### 2-Proteasome inhibitor (Bortezomib)

Bortezomib, a proteasome inhibitor, was first approved for the treatment of multiple myeloma. Preclinical studies have suggested a synergistic role of bortezomib with dexamethasone and additive effects to standard chemotherapy agents in acute leukemias.^125^ As a single agent, bortezomib did not produce durable responses in patients with relapsed/refractory ALL, despite demonstrable proteasomal inhibition.^126^ However, in a phase 2 study, bortezomib in combination with vincristine, dexamethasone, pegylated asparaginase and doxorubicin produced a response rate of 80% in children with relapsed/refractory pre-B-ALL.^127^ In a recent phase 2 COG trial, re-induction chemotherapy plus bortezomib resulted in a complete response in 68% of children with relapsed pre-B-ALL.^128^ Due to it’s ability to inhibit the NF-κB and NOTCH1 signaling pathways, bortezomib is being studied as frontline therapy in T-cell ALL. Recruitment is ongoing for a phase 3 trial of standard chemotherapy with or without bortezomib in children and young adults (age 2–30) with newly diagnosed T-cell ALL or T-cell lymphoblastic lymphoma (NCT02112916). In adults, recruitment has begun for a phase 2 trial of bortezomib with combination chemotherapy in relapsed/refractory ALL (NCT01769209).

### 3-JAK inhibitor (Ruxolitinib)

The JAK/STAT signaling pathway has been identified as a significant mechanism by which leukemic cells bypass normal growth and proliferation restrictions.^13^ In particular, Ph-like ALL appears to be dependent on JAK signaling. The most common rearrangements in Ph-like ALL involve the transmembrane receptor CRLF2, which signals through downstream JAK kinases. Many cytokine receptors, including IL-7 R, act through JAK kinases as well. In addition, JAK1 and JAK2 mutations are found in approximately half of CRLF2-rearranged Ph-like ALL.^12, 13, 14^ Preclinical studies have suggested benefit of ruxolitinib for the treatment of Ph-like ALL and CRLF2-rearranged ALL.^129, 130^ In addition, ruxolitinib inhibited tumor growth in *in vitro* and *in vivo* models of T-ALL with a gain of function in IL-7 R-alpha subunit.^131^ A phase 2 trial of ruxolitinib with standard multi-agent chemotherapy is currently open for recruitment of children, adolescents and adults with newly diagnosed high-risk B-ALL with CRLF2 rearrangements (NCT02723994).

### 4-Hypomethylating agent (Decitabine)

DNA methylation is an important epigenetic modification that regulates gene expression. It has long been reported that DNA methylation may play a role in the development of ALL and that methylation status may be used as part of risk stratification.^132, 133, 134, 135^ Decitabine is a cytosine analog that inhibits DNA methyltransferase by targeting it for degredation, thus causing hypomethylation of key regulatory domains on DNA. This leads to differentiation and suppression of tumor growth.^136^ Decitabine is currently approved for the treatment of myelodysplastic syndrome (MDS). In a case report, a young girl with her third-relapse of ALL was treated with a decitabine and dexamethasone regimen based on MDS dosing. She was able to undergo Allo-SCT after CR was achieved with re-induction therapy and remained in CR 8 months after transplant.^137^ In a MD Anderson phase I trial of decitabine for relapsed/refractory ALL, decitabine was shown to have efficacy when used in combination with Hyper-CVAD for re-induction therapy.^138^ In addition, decitabine monotherapy is well tolerated and thus offers a potential treatment option for relapsed disease in patients that cannot tolerate multi-agent chemotherapy. In a phase 2 study, decitabine and vorinostat (a histone deacetylase inhibitor) were given prior to vincristine, prednisone, PEG-L-asparaginase and doxorubicin for relapsed/refractory ALL.^139^ Results were promising with a CR rate of 50% (95% CI, 15.7–84.3%) and the OR rate 75% (95% CI, 34.9–96.8%). Decitabine has also been studied in preclinical trials of early T-cell precursor ALL (ETP-ALL), where it has been shown to be synergistic to conventional chemotherapy.^140^ Decitabine is currently being studied in the post-Allo-SCT setting (NCT02264873) and in combination with clofarabine, idarubicin and cytarabine for relapsed/refractory AML and ALL (NCT01794702).

### 5-PI3K/mTOR Inhibitors

The phosphatidylinositol 3-kinase/protein kinase B (PI3K/AKT) and mammalian target of rapamycin (mTOR) pathways are shown to be constitutively activated in 50–75% of T-ALL.^141^ Preclinical studies suggest that inhibition of the PI3K/AKT/mTOR pathways may be an effective treatment for T-ALL.^142, 143, 144, 145^ A dual PI3K/mTOR inhibitor, NVP-BEZ235, potently inhibited the proliferation ALL cells *in vitro*, causing G_0_/G_1_ arrest. Moreover, inhibition of proliferation was synergistic when NVP-BEZ235 was combined with cytotoxic agents.^144^ On the basis of this promising preclinical data, several clinical trials are underway to evaluate the use of mTOR and PI3K inhibitors in combination with multi-agent chemotherapy in the frontline and relapsed/refractory setting (NCT01756118, NCT02484430, NCT01523977, NCT01403415, NCT01614197 and NCT01184885).

### 6-Chimeric antigen receptor (CAR) T cells

Chimeric antigen receptor-modified (CAR) T cells are genetically engineered T cells that express the antigen-binding domain of an immunoglobulin linked via transmembrane domains to the intracellular T-cell receptor signaling moieties.^146^ This allows the T cells to recognize unprocessed antigens and to be activated in a major histocompatibility complex (MHC)-independent manner. First generation CAR-Ts contain intracellular signaling moieties derived only from the T-cell receptor/CD3 complex. In contrast, second- and third-generation CAR-Ts include co-stimulatory signals in the CAR gene constructs. More recently, fourth-generation CAR-Ts have been engineered to include a cytokine-expressing cassette.

The process of CAR T-cell therapy involves collecting T cells, introducing the CAR construct, and then an autologous transplant of the modified T cells back into the patient. Options for gene delivery methods include viral vectors and RNA-based methods.^147^ Using a viral vector has the benefit of inducing permanent gene expression and thus offering antitumor activity for as long as the transduced T cells persist. Theoretic risks of this method include malignant transformation of the engineered T cells if the CAR construct is inserted in such a way that it deregulates the expression of an oncogene.^148^ Another method of gene delivery involves direct transfer of an mRNA construct through electroporation.^149^ As no DNA is inserted into the genome of the T-cell, this eliminates the risk of malignant transformation. Given the high replicative potential of these T cells, this methods also offers the advantage of a profound antitumor response.^150^ However, the effects of direct mRNA insertion are transient and antitumor activity rarely persists beyond 7 days. Preclinical studies have suggested a role for RNA-based methods with multiple infusions; however, all current clinical trials utilize a viral vector to deliver the CAR construct.^150^

As mentioned above, CD19 is an ideal target for immunotherapy against B-cell ALL due to its near universal expression on B-lymphoblasts. In a pilot study at the Children’s Hospital of Philadelphia, Grupp *et al.*^151^ treated 53 children with relapsed/refractory ALL with lymphocyte depleting chemotherapy followed by CD19-directed CAR-Ts. A CR was observed in 50 patients (94%), with a 12-month EFS rate of 45% (95% CI, 31–66%) and OS rate of 78% (95% CI, 67–91%). The CAR-Ts were persistent at 6 months in 68% of the patients. Nearly all of the patients developed cytokine release syndrome (CRS). The 15 patients in which CRS was severe were effectively treated with the anti-IL-6-receptor antibody, tocilizumab.^152^ Important causes of treatment failure included the loss of circulating CAR-Ts and the expansion of a CD19-negative clone. CAR-Ts have also shown activity in adults with relapsed/refractory B-ALL. Davila *et al.*^153^ treated 16 adults at Memorial Sloan Kettering Cancer Center (MSKCC) with conditioning chemotherapy followed by CD19-directed CAR T-cell infusion. CR was observed in 88% of patients, with a 1–3 month persistence of CAR-Ts. Lee *et al.*^154^ reported a 66.7%% CR rate in a National Cancer Institute (NCI) intent-to-treat analysis of 20 children and young adults with ALL, with a median CAR-T persistence of 68 days. These data suggest a role for CAR-Ts in the treatment of relapsed/refractory ALL as a bridge to Allo-SCT or to produce durable remission. Limitations include the expansion of CD19-negative clones, the lack of long-term persistence of CAR-Ts after a single infusion, and the risk of CRS. Studies are ongoing to identify factors associated with the development of severe CRS and predict patients that would benefit from pretreatment.^155, 156^

Recently, the application of CAR-T cells has been expanded to CD22-positive B-ALL. Early preclinical studies have showed antitumor activity of CD22-directed CAR-Ts in *in vitro* and *in vivo* models that approximates that of CD19-directed CAR-Ts.^157^ Based on these findings, phase 1 trials using CD22-directed CAR-Ts are in the recruiting stages (NCT02650414). Preliminary results of nine patients have demonstrated that therapy is well tolerated and produced a sustained remission at 3 months in all three patients treated with a dose level of 1 × 10^6^ transduced T cells/kg.^158^

## Hematopoietic stem cell transplantation

After achieving complete response, treatment options include consolidation and maintenance chemotherapy or Allo-SCT for eligible patients. For high-risk patients and patients with relapsed/refractory disease, Allo-SCT has long been considered the standard of care and best chance for a durable response. While criteria differ between studies, in general high-risk disease is defined as Ph-positive ALL, elevated WBC count, CNS disease, high-risk gene rearrangements, or hypodiploidy. The LALA-94 and City of Hope and Stanford University series have shown a benefit of Allo-SCT over standard chemotherapy in these high-risk patients.^49, 159, 160^ It is therefore recommended that all high-risk young adults with an available donor undergo Allo-SCT during their first CR (CR1). Recent studies have suggested that patients with ETP-ALL and Ph-like ALL be treated as high-risk and be offered Allo-SCT during CR1 as well.^161, 162^ The role of Allo-SCT in standard-risk adults is less clearly defined. In general, MRD has emerged as a prognostic marker that can restratify patients to high-risk, making them candidates for Allo-SCT. Studies^32^ found that MRD-positivity is an independent risk factor for decreased relapse-free and overall survival. Subsequently, other studies^163^ evaluated the risk factors in patients treated with Allo-SCT versus standard chemotherapy after CR1. In patients with positive MRD, Allo-SCT was associated with improved relapse-free survival. However, in patients with a complete MRD response, there was no survival benefit to Allo-SCT over standard chemotherapy.^163^

Allo-SCT also should be considered in all patients that relapse, optimally after achieving a second CR (CR2). The LALA-94 trial showed a 5-year OS of 33% in patients who were able to undergo Allo-SCT during CR2 compared to 8% in patients who underwent Allo-SCT during active relapse.^164^ Patients who are unable to achieve CR2 by conventional methods should be considered for clinical trials with novel agents as a bridge to Allo-SCT. In the MRC/ECOG 2993 study, 5-year survival was highest in the group receiving a sibling donor Allo-SCT compared to unmatched donor or chemotherapy alone (23%, 16% and 4%, respectively).^165^

## Conclusion

Acute lymphoblastic leukemia has been touted as a major success story in pediatric oncology through the implementation of dose-intensification chemotherapy and Allo-SCT. However, due to high-risk disease characteristics and significant toxicity associated with chemotherapy in adults, outcomes are far less encouraging. There remains much uncertainty about how best to treat adults with ALL, as some studies have shown benefit of pediatric-inspired regimens. However, not all adults are able to tolerate such dose intensification and the exact subset of patients who are likely to benefit has not clearly been defined. Furthermore, elderly patients are particularly susceptible to the dose-limiting toxicities of these agents and are often excluded from Allo-SCT on the basis of performance status and medical comorbidities. Novel targeted therapies offer the promise of effective anti-leukemic activity with reduced toxicity from off-target effects. Given the diverse molecular and genetic alterations occurring in ALL, it is unlikely that a single agent will be effective for all patients with ALL. However, with the ability to characterize the immunophenotype and genotype of each patient’s leukemia, targeted therapy can be expected to lead to improvements in remission and survival as part of individualized treatment strategies. The successes from tyrosine kinase inhibition in CML have been translated to Ph-positive ALL, and second and third generation TKIs are being studied for use in high-risk Ph-like disease. Other signaling pathways, such as PI3K/AKT/mTOR pathway, are also promising targets for small molecule inhibition. In addition to targeting intracellular pathways, monoclonal antibodies recognize cell surface antigens. Immunoconjugates, such as inotuzumab ozogamicin, bind to leukemic cells, are internalized and release a cytotoxin that kills the leukemic cell; whereas dual-specific antibodies, such as blinatumumab, cause the direct activation of T cells against blasts. CAR-Ts involve a similar mechanism, in which a patient’s own T cells are genetically programmed to recognize leukemic cells, inducing an anti-leukemic immune response. Finally, existing agents, such as bortezomib, decitabine and ruxolitinib that are well tolerated in the treatment of various malignancies are now being studied for application in ALL. As the role of these novel agents is further defined and integrated into new treatment strategies, adult ALL may follow pediatric ALL as a major success story in the near future.

## Figures and Tables

**Table 1:** WHO classification of acute lymphoblastic leukemia^a^

B-cell lymphoblastic leukemia/lymphoma, not otherwise specified
*B-cell lymphoblastic leukemia/lymphoma, with recurrent genetic abnormalities*
B-cell lymphoblastic leukemia/lymphoma with hypodiploidy
B-cell lymphoblastic leukemia/lymphoma with hyperdiploidy
B-cell lymphoblastic leukemia/lymphoma with t(9;22)(q34;q11.2)[*BCR-ABL1*]
B-cell lymphoblastic leukemia/lymphoma with t(v;11q23)[*MLL* rearranged]
B-cell lymphoblastic leukemia/lymphoma with t(12;21)(p13;q22)[*ETV6-RUNX1*]
B-cell lymphoblastic leukemia/lymphoma with t(1;19)(q23;p13.3)[*TCF3-PBX1*]
B-cell lymphoblastic leukemia/lymphoma with t(5;14)(q31;q32)[*IL3-IGH]*
B-cell lymphoblastic leukemia/lymphoma with intrachromosomal amplification of chromosome 21 (iAMP21)^b^
B-cell lymphoblastic leukemia/lymphoma with translocations involving tyrosine kinases or cytokine receptors (‘BCR-ABL1–like ALL’)^b^^,14^

*T-cell lymphoblastic leukemia/lymphomas*
Early T-cell precursor lymphoblastic leukemia^b^

Abbreviations: ALL, acute lymphoblastic leukemia; WHO, World Health Organization.

a On the basis of The 2016 revision to the World Health Organization classification of myeloid neoplasms and acute leukemia.^23^

b Provisional entity.
